# Considerations for implementation of Alzheimer’s disease blood‐based biomarkers in clinical care based on recently updated NIA‐AA criteria

**DOI:** 10.1002/alz.088356

**Published:** 2025-01-09

**Authors:** Michelle M. Mielke

**Affiliations:** ^1^ Wake Forest University School of Medicine, Winston‐Salem, NC USA

## Abstract

**Background:**

The National Institute on Aging (NIA) and the Alzheimer’s Association (AA) recently updated their 2018 recommendations for the diagnosis and characterization of Alzheimer’s disease (AD), which include the use of blood‐based biomarkers (BBMs) for the differential diagnosis of AD. BBMs of amyloid‐beta, phosphorylated tau, neurodegeneration, inflammation, and alpha‐synuclein were defined, with a highlighted need for blood biomarkers of vascular brain injury. Many questions remain regarding how to implement these biomarkers for clinical care including diagnosis and to assess eligibility for disease modifying treatments (DMT).

**Method:**

Using examples from published and unpublished studies, the barriers to implementation of the use of AD BBMs will be synthesized. Additional discussion will focus on the current status and future discovery and implementation of non‐AD BBMs for vascular injury, inflammation, and alpha‐synuclein.

**Result:**

There are unique barriers and facilitators for the use of BBMs in primary and secondary care to diagnosis AD and assess DMT eligibility. Many questions remain regarding how to utilize the BBMs for disease staging in clinical care as compared to the research setting, which BBMs to use and when, as well as how to implement the BBMs at the population level.

**Conclusion:**

Advancing technology has led to the clinical use of BBMs to diagnose AD and assess treatment eligibility. Several considerations and steps are needed before these blood biomarkers can be implemented for use at the population level.

